# A comparison of machine learning approaches for the quantification of microglial cells in the brain of mice, rats and non-human primates

**DOI:** 10.1371/journal.pone.0284480

**Published:** 2023-05-01

**Authors:** Danish M. Anwer, Francesco Gubinelli, Yunus A. Kurt, Livija Sarauskyte, Febe Jacobs, Chiara Venuti, Ivette M. Sandoval, Yiyi Yang, Jennifer Stancati, Martina Mazzocchi, Edoardo Brandi, Gerard O’Keeffe, Kathy Steece-Collier, Jia-Yi Li, Tomas Deierborg, Fredric P. Manfredsson, Marcus Davidsson, Andreas Heuer

**Affiliations:** 1 Behavioural Neuroscience Laboratory, Department of Experimental Medical Sciences, Lund University Lund, Sweden; 2 Barrow Neurological Institute, Parkinson’s Disease Research Unit, Department of Translational Neuroscience, Phoenix, Arizona, United States of America; 3 Experimental Neuroinflammation Laboratory, Department of Experimental Medical Sciences, Lund University, Lund, Sweden; 4 Translational Neuroscience, College of Human Medicine, Michigan State University, Grand Rapids, MI, United States of America; 5 Brain Development and Repair Group, Department of Anatomy and Neuroscience University College Cork, Cork, Ireland; 6 Neural Plasticity and Repair, Department of Experimental Medical Sciences, Lund University, Lund, Sweden; Louisiana State University Health, Shreveport, UNITED STATES

## Abstract

Microglial cells are brain-specific macrophages that swiftly react to disruptive events in the brain. Microglial activation leads to specific modifications, including proliferation, morphological changes, migration to the site of insult, and changes in gene expression profiles. A change in inflammatory status has been linked to many neurodegenerative diseases such as Parkinson’s disease and Alzheimer’s disease. For this reason, the investigation and quantification of microglial cells is essential for better understanding their role in disease progression as well as for evaluating the cytocompatibility of novel therapeutic approaches for such conditions. In the following study we implemented a machine learning-based approach for the fast and automatized quantification of microglial cells; this tool was compared with manual quantification (ground truth), and with alternative free-ware such as the threshold-based ImageJ and the machine learning-based Ilastik. We first trained the algorithms on brain tissue obtained from rats and non-human primate immunohistochemically labelled for microglia. Subsequently we validated the accuracy of the trained algorithms in a preclinical rodent model of Parkinson’s disease and demonstrated the robustness of the algorithms on tissue obtained from mice, as well as from images provided by three collaborating laboratories. Our results indicate that machine learning algorithms can detect and quantify microglial cells in all the three mammalian species in a precise manner, equipotent to the one observed following manual counting. Using this tool, we were able to detect and quantify small changes between the hemispheres, suggesting the power and reliability of the algorithm. Such a tool will be very useful for investigation of microglial response in disease development, as well as in the investigation of compatible novel therapeutics targeting the brain. As all network weights and labelled training data are made available, together with our step-by-step user guide, we anticipate that many laboratories will implement machine learning-based quantification of microglial cells in their research.

## Introduction

Neuroinflammation is a complex multiphase response occurring within the central nervous system (CNS) that involves different types of immune cells (e.g. astrocytes, microglia, endothelial cells, peripherally derived immune cells), and is mediated by the production of various primary and secondary factors (e.g., cytokines, chemokines, reactive oxygen species) [1]. Microglial cells represent a small fraction of specialized tissue-resident macrophages of the brain, carrying out various functions during both development and adulthood [2]. In adult organisms, microglial cells are well known for constantly surveying the CNS [3] and protecting it against pathological insults (e.g., brain injury, pathogen infiltration, proteins aggregation, neurodegenerative diseases) [4]. Microglial cells are generally rapidly activated upon minimal disruptive changes in the CNS, making them the first line of defence against any potential deleterious stimulus [5–8]. In physiological conditions, microglial cells interact with the local microenvironment and with surrounding cells presenting a resting ramified morphology with long processes extended in the proximal area [9]. When activated, microglial cells undergo specific modifications that include changes in their migration and proliferation rates (fast recruitment on the site of insult and in the near proximity) [10], changes in their morphology (from resting ramified to a rod-like shape with hypertrophic process to an ultimate phagocytic, ameboid shape with abundant cytoplasm and short processes) [6, 11, 12], changes in antigen presentation [13, 14] and production/release of various inflammation-mediating factors [13, 15–18]. Neuroinflammation is a common feature of many of neurodegenerative diseases, such as Parkinson’s disease (PD), Alzheimer’s disease (AD), multiple sclerosis (MS) [19–27] and post-mortem immunohistochemistry-based (IHC) quantification of neuroinflammation—specifically of microglial cells—is crucial in the investigation of neurodegenerative diseases of human brain tissue as well as preclinical animal model. We have previously applied manual/semiautomated quantification and evaluation of microglial morphology in several rodent models of stroke [28] and neurodegenerative diseases [29, 30] as well as in human brain sections [31]. The most common methods are based on qualitative anatomopathological evaluation of cell morphology (e.g., resting vs activated status), area occupied by cells (e.g., threshold-based cell density), area/perimeter ratio (e.g., Scholl-based analysis), and cell number quantifications (number of cells in the target area) using either ImageJ, proprietary software/scripts or manual procedures [32–42]. Many of these methods are time consuming, error prone, require experienced users, and are very likely to introduce experimenter bias.

In this work, we developed and compared machine learning (ML)-based approaches for the fast and automatized recognition and quantification of microglial cells in post-mortem tissues using three different detector networks. Furthermore, we validated the ML approaches by comparison to manual quantifications (ground truth), as well as threshold-based quantifications (Fiji), and Ilastik—an open-source machine-learning based software for image classification and segmentation. After training, the three different neural networks (Faster R-CNN, RetinaNet and YOLOv3), were validated on various animal species in our preclinical rodent models of PD and AD—two neurodegenerative disorders where neuroinflammation is associated with the disease progression. We demonstrate that ML based automated quantification is capable of recognition and quantification of microglial cells with equal accuracy to manual quantifications performed by a trained researcher. The code and weights of the respective networks have been made available in the GitLab repository where they can be modified to the individual researchers needs. We believe this new approach offers new tools to study microglia alterations and facilitate more homogenised comparisons to be made between results among researchers studying microglia in brain diseases.

## Materials and methods

In the current manuscript we trained three different neural networks (Faster R-CNN, RetinaNet, YOLOv3) on rat and non-human primates (NHP) tissues. We subsequently validated the performance of the models against the ground truth (manual quantifications) and compared the performance to the open-source software Fiji (ImageJ) and Ilastik on brain tissues obtained from a PD rat model, an AD mouse model, and wild type NHP tissue. The study is reported in accordance with ARRIVE guidelines.

### Animals

#### PD model (rats)

All the surgical and experimental procedures were designed, approved, and performed in accordance with the EU directive for the use of animals in research (2010/63/EU), approved by the local ethical committee and registered with the Swedish Department of Agriculture (Jordbruksverket). Female Sprague-Dawley rats (Janvier–Germany) were kept under a 12:12 hours light/dark cycle, constant temperature of 21° C, 50% humidity and *ad libitum* access to water and food. The rats were acclimatized to the new environment for 5 days before any experimental procedure.

#### AD model (mice)

All the experimental procedures were designed, approved, and performed in accordance with the EU directive for the use of animals in research (2010/63/EU), approved by the local ethical committee (Dnr 5.8.18-01107/2018) and registered with the Swedish Department of Agriculture (Jordbruksverket). Transgenic 5xFAD mice with C57/BL6-SJL background, and age-matched mice as controls (WT) (Jackson Laboratory—USA) were kept under a 12:12 hours light/dark cycle, constant temperature of 21° C, 50% humidity and *ad libitum* access to water and food. Mice were acclimatized to the new environment for few weeks before any experimental procedure. The 5xFAD mice carried three mutations in the human APP transgene (the Swedish mutation, K670N/M671L; the Florida mutation, I716V; and the London mutation, V717I) and two mutations in the human PSEN1 transgene (M146L/L286V). These mutations are related to familial forms of AD, and the transgenes are expressed under the neuron-specific Thy-1 promoter.

### NHP

Archived control brain tissue from 3 middle aged (12 years old) African green non-human primates (Chlorocebus aethiops) was used for this study. Tissue harvest was performed in accordance with RxGen Institutional Animal Care & Use Committee. All NHP *in vivo* procedures using adult male, either colony-born or ethically sourced from the population on St. Kitts, African green monkeys (AGMs; *Chlorocebus sabaeus*) were conducted by Virscio, Inc. at the AAALAC accredited St. Kitts Biomedical Research Foundation, St. Kitts, West Indies. Institutional Animal Care and Use Committee approval was in full compliance with the National Research Council (US) Committee for the Update of the Guide for the Care and Use of Animals, facility standard operating procedures, and in accordance with AAALAC standards for the use of animals in biomedical research. NHPs were housed in standard nonhuman primate cages with *ad libitum* access to water and fed primate chow (Enviro Teklad 8773) supplemented with local fruits and vegetables. Animals were provided environmental enrichment toys to promote psychological wellbeing which was assessed twice daily by cage side observation by observers trained to monitor AGM behavior and general health. The number of food biscuits consumed daily was qualitatively monitored at these times as well. Prior to necropsy, monkeys were sedated with ketamine (8–10 mg/kg, IM) and euthanized with sodium pentobarbital (100 mg/kg, IV).

### *In vivo* experimental procedures

#### AAV production

AAV9-CBA-aSYN/aSYN (AAV-aSYN) and AAV9-CBA-noTG (AAV-noTG) viruses were produced using chloroform extraction [43, 44]. HEK293T cells were triple transfected with ITR-transgene, pAAV2/9n and the helper plasmid pXX6 using PEI. AAVs were harvested 72h post-transfection using polyethylene glycol 8000 (PEG8000) precipitation and chloroform extraction followed by PBS exchange in concentration columns. AAVs were titered using droplet digital PCR (ddPCR) [45, 46], with primers specific for the ITRs (forward primer 5′-CGG CCT CAG TGA GCGA-3′ and reverse primer 5′-GGA ACC CCT AGT GAT GGA GTT-3′), and then normalized to a working titer of 2.5x10^12^ genome copies (gc)/ml using modified Phosphate Buffer Saline (PBS) Mg^++^/Ca^++^.

#### PD model (rats)

The preclinical rodent model of PD used in the present study is based on adeno-associated viral vector (AAV9) overexpression of human WT alpha synuclein (h-aSYN) as previously described [47]. As control, we used an identical construct lacking the transgene (noTG), as described previously [47]. Prior to AAV delivery, animals were anesthetised with a mixture of 5% isoflurane and oxygen and placed into a stereotactic frame. Injections were performed using a pulled glass capillary connected to a 10 μl Hamilton syringe [48]. 2 μl of virus were unilaterally injected into the substantia nigra (SN) at two different sites (in mm, from Bregma): 1) A/P: -5.3; M/L: -1.8; D/V: -8; and 2) A/P: -5.6; M/L: -2.5; D/V: -7.5, with a speed of 0.5 μl/min. Once the delivery was completed, the needle was left in place for additional 4 minutes to allow for diffusion.

### Immunohistochemistry (IHC)

#### PD model (rats)

Immunohistochemistry was performed as described previously [49]. In brief, 12 weeks after the lesion, rats were sacrificed with an intraperitoneal injection of sodium pentobarbital (1 mg/kg) and transcardially perfused with 50 ml 0.9% saline solution, followed by 250 ml of 4% paraformaldehyde (PFA) in phosphate buffered saline (PBS) solution (pH = 7.4). The brains were extracted and placed for 24 h post-fixation in PFA at 4°C and transferred into 25% sucrose until sunk. All brains were sectioned into 40 μm coronal 1:12 series using a freezing microtome (SM200R, Leica) and stored in antifreeze solution at -20° C until use. DAB-immunohistochemistry (DAB-IHC) was performed as described previously [47]. Briefly, the sections were removed from the anti-freeze solution and washed 3 times in PBS (pH = 7.4), and subsequently incubated for 15 minutes in PBS with 3% H_2_O_2_ and 10% methanol to quench endogenous peroxidase activity. Brain slices were then washed 3 times in PBS and incubated for 1 hour at room temperature (RT) in a blocking solution (5% goat serum and PBS-T (PBS containing 0.25% triton X-100)) and then transferred into the primary antibody (IBA1, Rabbit, 1:1000 –WAKO 019–19741) in 5% serum and incubated overnight at 4°C with gentle agitation. The following day, the sections were washed 3 times in PBS and incubated for 1 hour at RT in blocking solution, followed by incubation in secondary antibody (Anti-rabbit biotinylated, Goat, 1:200 –Vector Laboratories BA6000) in 5% serum for 1 hour at RT. The sections were then washed 3 times in PBS and incubated for 1 hour with the ABC complex (ABC, Vector Laboratories) following the vendor’s protocol. After ABC incubation they were washed 3 times in PBS and the colour reaction was produced by incubation in DAB substrate (DAB, Vector Laboratories) with H_2_O_2_. Immunolabelled sections were washed in PBS and mounted on gelatine-coated glass slides, airdried overnight, and then dehydrated for 5 minutes in increasing series of ethanol (2x 70%, 2x 95%, 2 x 99.5%) and lipids were removed through 2x 5-minute incubation in Xylene before cover-slipping with DPX mounting medium (Sigma).

#### AD model (mice)

Mice were deeply anesthetized using pentobarbital and perfused with saline, followed by 4% paraformaldehyde (PFA) fixation. Brains were post-fixed in the same fixative overnight and then immersed in PBS with 30% sucrose until use. Brains were cut into 20 μm thick sections using a microtome (Leica SM2010R) and stored at -20° C in an anti-freeze solution. DAB-IHC was performed as previously described in the PD model (rats).

#### NHP

Animals were deeply sedated and euthanized by transcardial saline perfusion. Following removal, brains were post-fixed with 4% formaldehyde for 48–72 hours and thereafter transferred to a sucrose gradient. Sections were coronally cut with a thickness of 40 μm and stored in cryoprotectant. IBA1 IHC was performed as previously described [50]. Briefly, tissue was quenched in peroxide, and following a blocking step, incubated with the primary antibody (Wako, Cat#019–19741) for 2 days at 4° C, and then treated with the Vector ABC detection kit. Development was done in 0.5 mg/mL 3,3′ diaminobenzidine and 0.015% hydrogen peroxide in Tris buffer. Sections were mounted on subbed slides, dehydrated with increasing concentrations of ethanol followed by Xylene, and coverslipped with cytoseal.

### Image acquisition

Quantification of microglia was performed on one image of the central SN of the ipsilateral and contralateral hemisphere (for PD model) and on one image of the central SN of the right hemisphere for the AD model and NHP. For rats and mice, images were acquired in RGB using a Leica DMI8 inverted microscope with a 20x objective and a z-step size of 1 μm though the entire thickness of the tissue. Images were either saved as a multi-stack or processed to extended depth of field (EDF) and then saved. Acquisition of NHP images was carried out using a Nikon Eclipse Ni upright microscope provided with a 20x objective. Each image consisted of 30 μm thick z-stack of a single image-tile sized to match the mice and rats’ files acquired with a z-step of 1 μm.

### Neural networks

#### Deep learning network architectures

We utilized a transfer learning approach where we take weights of a pretrained model which was trained on a large dataset were used to enhance the performance of model training on a small dataset. For training of the object detection models, we used the ResNeXt-101 [51] and Darknet-53 [52] backbone architectures which were trained on the ImageNet and the COCO dataset, respectively.

#### Faster R-CNN

**Fig 1:**
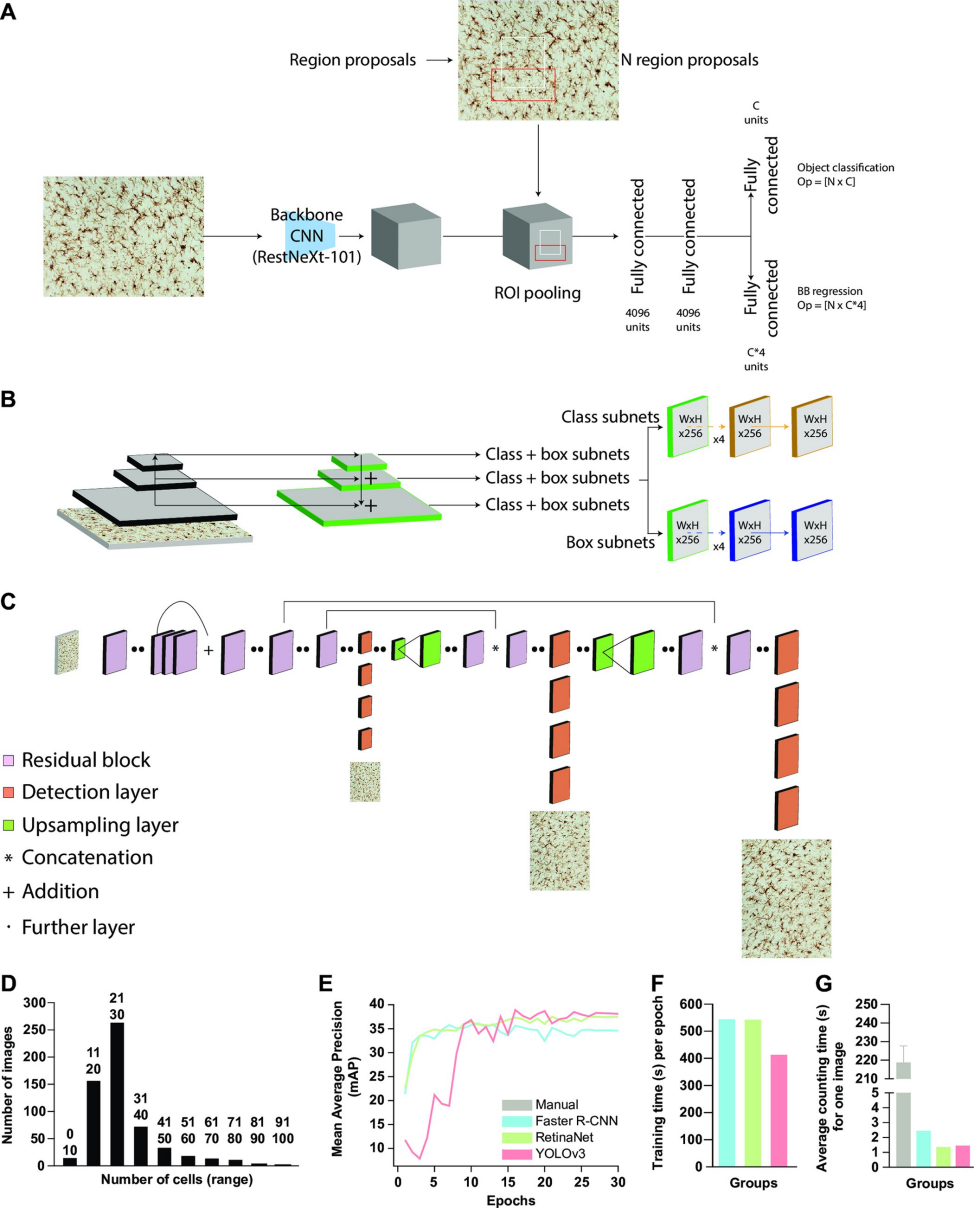
AI network overview and AI network characterization. **A:** Schematic representation of Faster R-CNN. **B:** Schematic representation of RetinaNet. **C:** Schematic representation of YOLOv3. **D:** Overview of the distribution of IBA1^+^ cells detected in the analysed images. **E:** Curve of mean Average Precision values in the test set versus epoch number. **F:** average time (s) for manual quantification, Faster R-CNN, RetinaNet and YOLOv3.

Faster R-CNN (Fig 1A) [53] belongs to the family of region based convolutional neural network models. It is a two-stage architecture, consisting of a Region Proposal Network (RPN) to generate ROIs with a high chance of containing objects and a detection network to classify and localize objects. The ResNext-101 architecture is used as a feature extractor generating feature maps by performing convolution operations on the input image. The resulting feature maps are fed into the RPN. Non-Maximum Suppression takes the RPN’s generated region of interest and reduces the number of bounding boxes by removing the boxes which are overlapping based on probability of containing object and area of overlap. The resulting feature map generated by the feature extractor and resulting bounding box of the RPN are fed forward into the detection network. To deal with non-uniform size of the feature map, a ROI pooling layer is added to scale and crop the feature map before feeding it to the detection network. The detection network consists of a fully connected network and is followed by a classification layer and a bounding box regression layer. The classification layer returns the class probability, and the regression layer returns the coordinates of the bounding box.

#### RetinaNet

RetinaNet (Fig 1B) [54] is a single-stage object detector a with feature pyramid network to detect small and dense objects. In a single stage architecture, the training procedure is dominated by easily classified background examples and it leads to the learning of foreground examples difficult. RetinaNet uses the “Focal Loss” loss function to improve performance by biasing the training towards more difficult examples. The RetinaNet architecture consists of a backbone network and is followed by two task specific subnetworks for bounding box regression and classification. The backbone network has a ResNeXt-101 [51] for feature extraction and a “Feature Pyramid Network” (FPN) [55] on the top of ResNeXt-101 for detecting objects of different scale and sizes.

#### YOLOv3

YOLOv3 [52] is an improved version of YOLO (Fig 1C). It is a single stage architecture, which gives the class probability as well as the objects’ location. Darknet-53 is used as a feature extractor based on convolutional layers. YOLOv3 consists of a stack of 106 convolutional layers. YOLOv3 detects objects at three different scales with the help of a detection kernel on feature maps of three different sizes (Fig 1C). The shape of the detection kernel is 1 x 1 x (B x (5 + C)), where B is the number of bounding boxes a cell on the feature map can predict, 5 is for the 4 bounding box attributes and one object confidence, and C is the number of classes. The detection kernels are added at the 81^st^, 94^th^ and 106^th^ layers to detect the object at different scales. The resulting feature maps after 81^st^, 94^th^ and 106^th^ layers are responsible for detection of small, medium, and large size objects, respectively.

#### Dataset

The initial dataset was divided into a training (n = 602), test (n = 100) and validation (n = 102) sets. Bounding boxes were created manually using the roboflow online annotation tools and exported to coco format for subsequent training of the models. The final dataset contained 21,363 total cells with an average of 26.6 cells per image and the majority of images contained between 12 and 41 cells (Fig 1D). The following training script, which can be used for retraining, can be found in the following link: https://colab.research.google.com/drive/1FdFHsVhTU1-Xej8WIQiHqXTcVBJrmges?usp=sharing.

The training and validation datasets were organized as following described in Table 1.

**Table 1:** Overview of the images used for training dataset and validation dataset for the algorithms.

Species	Model	Location	Hemisphere	N of images for training	N of images for validation
Rat	PD	SN	Ipsilateral, Contralateral	521	90
NHP	WT	SN	Right	81	12
**Total N**				**602**	**102**

#### Robustness

To further validate the robustness of our model we asked collaborators from 3 different laboratories (Barrow Neurological, BN; Michigan State University, MSU; University College Cork, UCC) to test the CNNs on images taken with their respective IHC protocols and microscopes (see Table 2). Furthermore, we downloaded Fluorescent IHC images from an online database (https://github.com/tkataras/ACCT-Data-Repository [56]). The images (grayscale, PNG, 2–3 focal layers/image) were downloaded and processed in Fiji. We first generated a stack from the focal layers and subsequently generated EDF images (Sorbel Projection) using the CLIJ2 plugin (https://clij.github.io).

**Table 2:** Overview of images used for robustness testing.

University	Species	IHC	Objective	Microscope	Images	Parameters
UCC	Rat	DAB Iba 1	20x	Olympus BX53-U	10	Threshold: 0.5 NMS: 0.5
MSU	Rat	Ni-DAB Iba1	20x	Nikon Eclipse 80i	14	Threshold: 0.1 NMS: 0.5
BN	Rat	DAB Iba 1	20x	Nikon Eclipse Ni-U	9	Threshold: 0.1 NMS: 0.1
Gitlab	Mice	FITC-Iba1	10x	Zeiss 200 M	24	Threshold:0.1 NMS: 0.5

UCC = University College Cork, MSU = Michigan State University, BN = Barrow Neurological.

To demonstrate the best performance parameters of our algorithm we generated EDF stacks at different exposures (25 ms to 400 ms) to utilize different dynamic ranges of the camera when producing the images.

To demonstrate the best performance parameters of our algorithm we generated EDF stacks at different exposures (25 ms to 400 ms) to utilize different dynamic ranges of the camera when producing the images.

#### Download

The weights of the respective models as well as the annotated code can be downloaded from our Gitlab repository (https://gitlab.com/cell-quantifications/Microglia). The annotated images used in the present study for training and validation are available online (https://www.kaggle.com/datasets/bnllund/microglia).

### Quantifications

#### Comparison to other methods

We compared the performance of the networks by manual quantification, as well as by other available tools as following described in Table 3.

**Table 3:** Overview of the quantified images and validation of the algorithms with other methods.

Species	Model	Group	Location	Hemisphere	Total N of quantified images	Quantification tools
Rat	PD	AAV-noTG	SN	Ipsilateral	6	Manual Faster R-CNN RetinaNet YOLOv3 Ilastik FIJI
		AAV-aSYN		Contralateral	6	
Mouse	AD	WT		Right, Left	10	
		5xFAD			4	
NHP	WT	WT		Right	8	

Abbreviations: NHP: Non-Human Primate, PD: Parkinson’s disease, AD: Alzheimer’s disease, WT: Wild-type, AAV: Adeno Associated Virus, noTG: no transgene, aSYN: alpha-synuclein, 5xFAD: Alzheimer model with 5 AD linked mutations, SN: substantia nigra.

### AI models

The detectron-2 framework was used for model building and assigning parameters of Faster R-CNN and RetinaNet. The stochastic gradient descent (SGD) method was used to train all three architectures with varying learning rates. By applying a small penalty to the loss function, typically the L2 norm of the weights, the optimizer with a weight decay of 0.0005 ensured a regularization technique in Faster R-CNN and RetinaNet. At the time of inference, the probability threshold value was kept 0.5 for all the models. All three architectures used the stochastic gradient descent method with momentum. We trained for 32 Epochs in batch sizes of 4 with a learning rate of 0.001.

#### Ilastik

Ilastik is an open-source machine learning-based software for interactive image classification, segmentation, and analysis *(**https*:*//www*.*ilastik*.*org/index*.*html**)* [57]. IBA1^+^ cell quantification was performed using the Pixel-classification tool, after a training of the algorithm performed on 5 images.

#### Fiji

Microglia quantification using Fiji was performed using multi-stack images acquired as described previously [58]. Briefly, images were imported as a 3D stack and an appropriate threshold was applied and the number of 3D IBA1^+^ immunoreactive structures was obtained. A microglial cell was defined as an IBA1^+^ immunoreactive structure with a size larger than 20 μm and a circularity of 0.25 to 1 [58]. The results are expressed as the total number of IBA1^+^ cells in the quantified image.

#### Manual quantification

Manual quantification of microglial cells was performed on the same images used for AI-based quantification. All IBA1+ cells in the image were quantified using the ImageJ cell counter tool by two experimenters blinded to the experimental group.

### Statistics

All data were analysed using IBM-SPSS V.26 with a statistical alpha set at 0.05. For multigroup comparison, one-way ANOVA was followed by Bonferroni *post-hoc* test. Data are expressed with SEM error bars. Significance is displayed in comparison to the manual quantifications. For correlation analysis we used linear (r coefficient of Spearman) regressions with a significance level set at α = 0.05. Levels of significance are denoted in the Figures as * p < 0.05; ** p < 0.01, and *** p < 0.001.

## Results

All three architectures were trained on the training set data and evaluated against the manually annotated test set.

**Fig 2:**
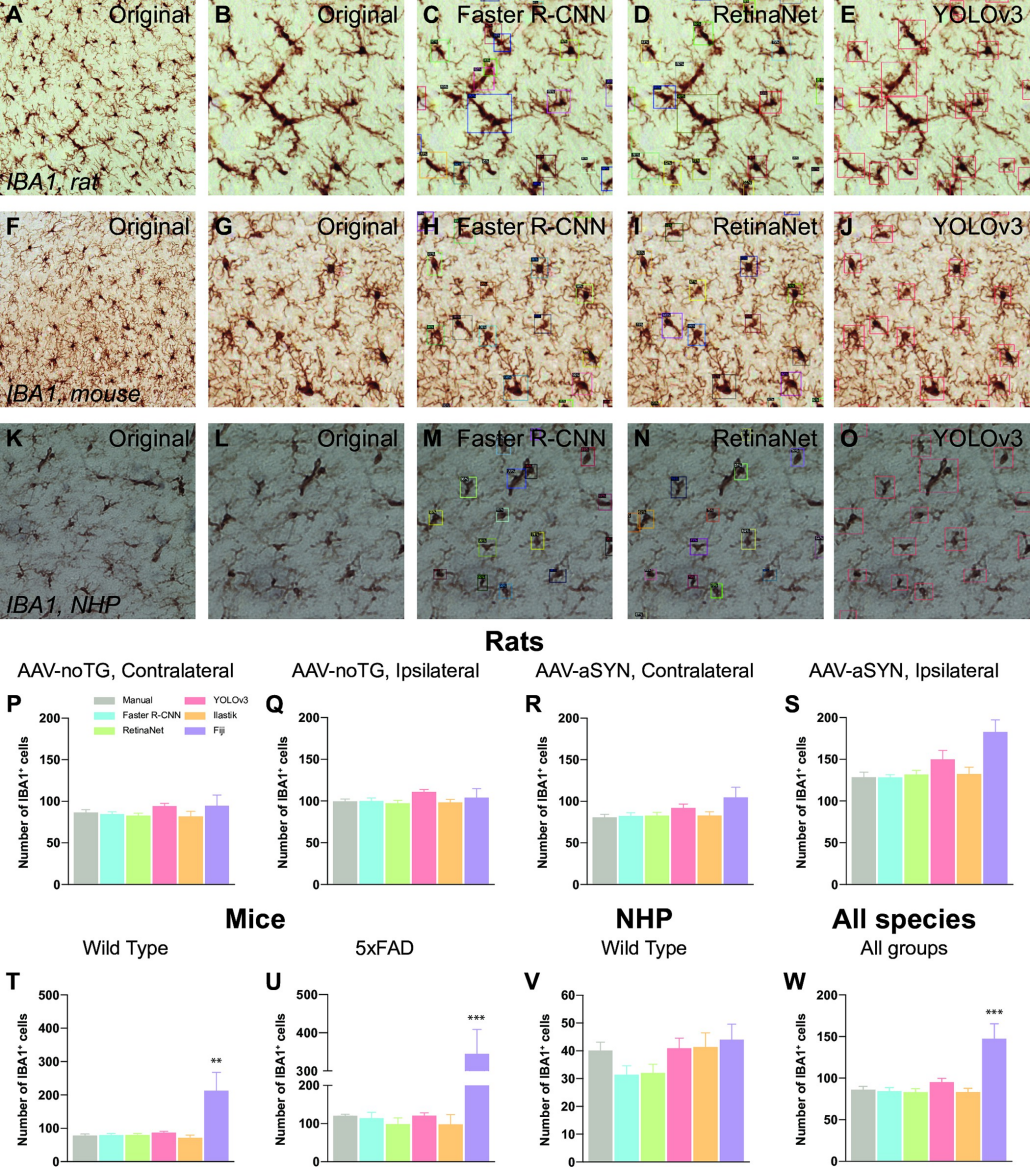
Quantification analysis of IBA1^+^ cells in three different mammalian species. **A:** Source image of rat SN immunolabelled for IBA1^+^ microglial cells. **B:** Closeup of image A. **C-E**: output of AI-based quantification using Faster R-CNN, RetinaNet, and YOLOv3, respectively. **F:** Source image of mouse SN immunolabelled for IBA1^+^ microglial cells. **G:** Closeup of image F. **H-J**: output of AI-based quantification using Faster R-CNN, RetinaNet and YOLOv3, respectively. **K**: Source image of NHP SN immunolabelled for IBA1^+^ microglial cells. **L:** Closeup of image K. **M-O**: output of AI-based quantification using Faster R-CNN, RetinaNet, and YOLOv3 respectively. For all images, each bounding box represents the detected, and therefore quantified cell. **P-S:** Comparison of the different quantification methods for rats injected with the control vector on the contralateral (P) and ipsilateral side of injection (Q) as well as for rats injected with the vector overexpressing h-aSYN on the contralateral (R) and ipsilateral (S) side of injection. **T, U:** Comparison of the different quantification methods for the IBA1^+^ cells in the SN of WT (T) and 5xFAD mice (U). **V:** Comparison of the different quantification methods for the IBA1^+^ cells in the SN of WT NHP. **W:** Comparison of the different quantification methods for the IBA1^+^ cells in all the three analysed species. Data are expressed as mean ± SEM; *** = p < 0.001.

For the evaluation of the model, the mean Average Precision (mAP) and Average Precision scores at 0.75 (AP_75) and 0.05 (AP_50) Intersection of Union (IoU) thresholds were taken into consideration. The mean Average Precision (mAP) score is calculated by taking the mean average precision over all the classes and/or overall Intersection of Union (IoU) thresholds. Fig 1E displays the curve of mAP values in the test set versus epoch number. After 30 epochs, the mAP approximates the horizontal asymptote (Fig 1E). The mean Average Precision (mAP) score of the respective architectures was Faster R-CNN = 0.36, RetinaNet = 0.38, and YOLOv3 = 0.39, respectively. AP_75 and AP_50 measure the average precision of a model when the predicted bounding box overlaps with the ground truth bounding box by at least 75% and 50% respectively (see Table 4). Faster-RCNN is able to correctly detect and localize 12.79% and 88.89% of the objects in the image with an IoU overlap of at least 0.50 and 0.75 between the predicted and ground truth bounding boxes. RetinaNet localizes 14.79% and 90.61% of objects correctly at IoU overlap of 0.50 and 0.75 between the predicted and ground truth bounding boxes and YoLOv3 localizes 13.87% and 89.27% correctly at IoU overlap of 0.50 and 0.75 between the predicted and ground truth bounding boxes. RetinaNet localizes the objects better than our other implementation at IoU overlap of 0.50 and 0.75 between the predicted and ground truth bounding boxes. Training time per epoch was similar between the three networks with Faster R-CNN = 541 seconds, RetinaNet = 539 seconds, and YOLOv3 = 410 seconds (Fig 1F). The three architectures were trained on rat and NHP tissues. To assess the robustness of the respective algorithms, we quantified microglial cell numbers on specimens from three different mammalian species–rats, mice, and NHP, respectively. Using a Windows laptop fitted with a AMD Ryzen 7 4800H 2.90 GHz processor, 16 GB RAM, and Nvidia RTX 2060 graphic processing unit (GPU), each image was quantified with an average time of 1–3 seconds (s) per image (Faster R-CNN = 2.5 seconds; RetinaNet = 1.4 seconds; YOLOv3 = 1.5 seconds), whilst the average time for manual quantification was 220 s ± 9.11 (Fig 1G). However, it is important to mention that the time taken by each model cannot be always constant, since it is directly influenced by PC proprieties, activity running at the same time of analysis, as well as network performance. Overall, all the three AI-based tools performed similarly in detecting microglial cells in rats (Fig 2A–2E; 2P–2S), mice (Fig 2F–2J; 2T, 2U) and NHP (Fig 2K–2O; 2V), when compared to manual quantifications. We first evaluated whether an overall difference between the quantification methods was detectable among all the analysed specimens (Fig 2W); we observed statistical differences within the methods (F_5,270_ = 8.47, p < 0.001), and Bonferroni post-hoc analysis revealed a significant difference between manual and Fiji-based quantification (p < 0.001), but neither between manual quantifications nor any other method (all *t* < 0.71, *p* = n.s.).

**Table 4:** Average Precision (AP) score at different IOU threshold.

AI network	AP_75_	AP_50_
Faster R-CNN	12.79	88.89
RetinaNet	14.79	90.61
YOLOv3	13.87	89.27

### Rats

Overexpression of h-aSYN via AAV vector in the SN of rats is a widely used preclinical rodent model of PD. The overexpression of h-aSYN is known to induce a neuroinflammatory response in the area of injection (39, 47). Quantification of IBA1^+^ cells in the contralateral (F_5,30_ = 0.653, p = n.s.) and ipsilateral (F_5,30_ = 0.753, p = n.s.) SN of rats receiving delivery of an AAV-noTG did not show any significant difference between the methods (Fig 2Q and 2R), with an overall quantified number of IBA1^+^ cells of 88.1 ± 5.95 and 102.4 ± 5.18 respectively. Similarly, we did not observe statistical difference between the quantification methods in either the contralateral (F_5,30_ = 5.33, p = n.s.) or ipsilateral (F_5,30_ = 1.85, p = n.s.) SN of rats receiving delivery of the AAV-h-aSYN (Fig 2S and 2T), with an overall number of quantified cells in the contralateral hemisphere of 88.3 ± 6.11, and 143.1 ± 8.44 in the ipsilateral hemisphere. Interestingly, in the ipsilateral hemisphere of the rats injected with AAV-h-aSYN the Fiji-based quantification returned a slightly higher number of cells when compared with other methods, indicating skewed results using threshold-based methods; Bonferroni post-hoc analysis however did not reveal any significant difference when compared with the manual quantification.

### Mice

5xFAD mice are a well characterized transgenic mouse line which is widely used as a model of AD and AD related phenotypes, presenting early intracellular amyloid β (Aβ), astrogliosis, synaptic degeneration, neuronal loss, and impaired behavioural phenotype, which we have used in various experimental studies [30, 31, 59–61]. Quantification of nigral IBA1^+^ cells was performed in 6-months old WT mice as well as in 5xFAD transgenic mice. Quantification of IBA1^+^ cells in the WT group (Fig 2U) revealed a significant difference between the quantification methods (F_5,54_ = 5.33, p < 0.001), and Bonferroni post-hoc analysis returned a statistical difference (p < 0.01) between the manual quantification and the Fiji-based quantification. Similarly, the same trend was seen in the 5xFAD group of mice (Fig 2V), with an inter-method difference (F_5,18_ = 9.86, p < 0.001), and a significant difference between the manual and Fiji quantification (p < 0.001). Except for the Fiji-based quantification, the numbers of IBA1+ cells in both the WT (103.6 ± 14.9) and 5xFAD (151.4 ± 23.3) animals are similar between all the quantification tools.

### NHP

NHP are frequently utilised in preclinical research due to their close phytogenic relationship with humans and their physiological similarities [62–64]. Quantification of microglia from naïve NHP midbrain tissues (Fig 2W) revealed similar results among all the quantification methods with an overall detected number of IBA1^+^ cells of 38.5 ± 4.11 (F_5,42_ = 1.55, p = n.s.). Interestingly, in this condition Faster R-CNN and RetinaNet slightly underestimated the number of IBA1+ cells when compared to the manual quantification, YOLOv3, Ilastik or Fiji; however, such difference was not statistically significant.

### Correlation

**Fig 3:**
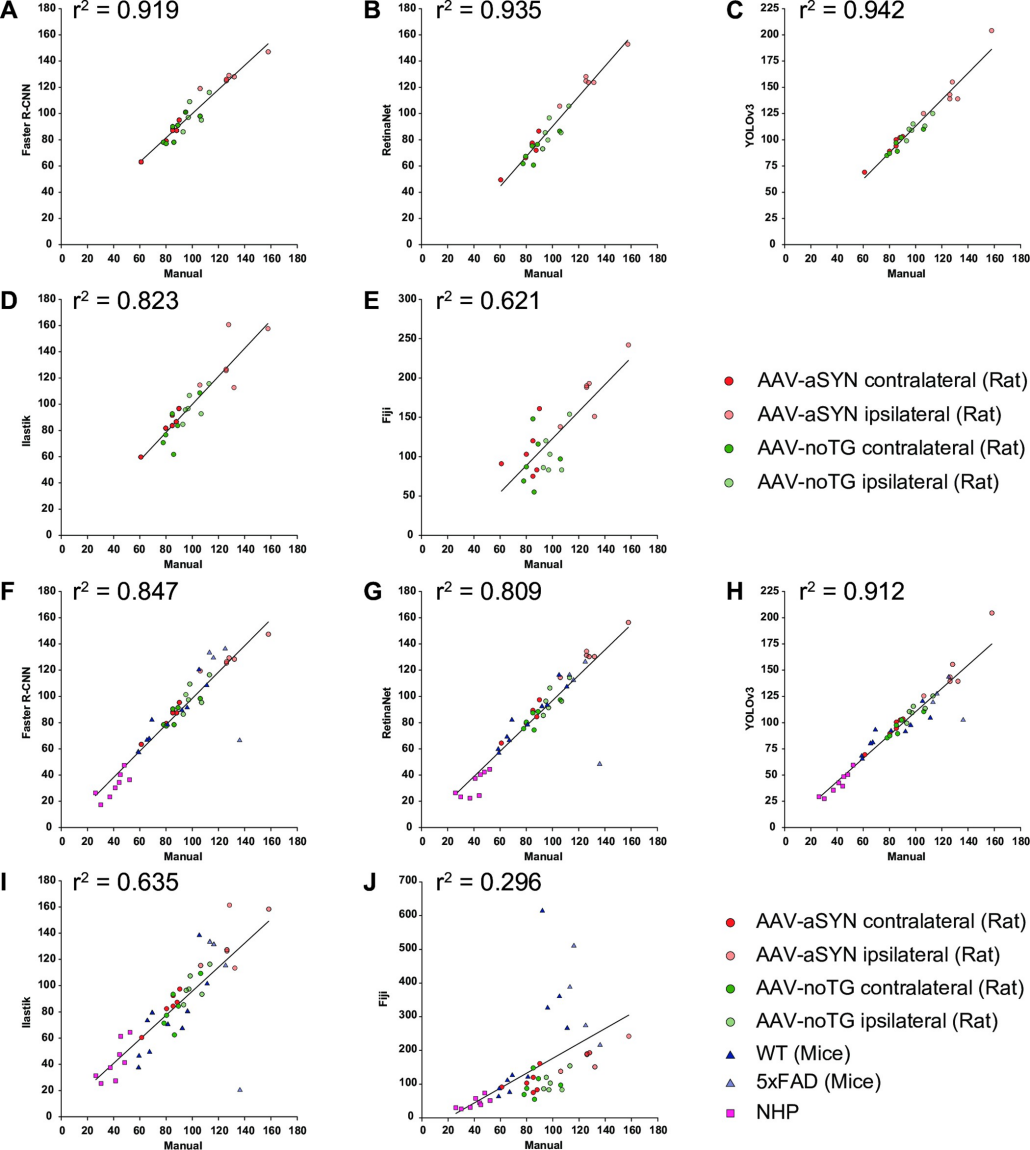
Correlation analysis of the different quantification methods. Correlation analysis for the AAV-noTG and AAV-aSYN (both ipsilateral and contralateral hemisphere) groups comparing manual quantification vs A: Faster R-CNN; B: RetinaNet; C: YOLOv3; D: Ilastik; E: Fiji. Correlation analysis for the AAV-noTG, AAV-aSYN rat group (both ipsilateral and contralateral hemisphere), WT and 5xFAD mice groups and WT NHP group comparing manual quantification vs **F**: Faster R-CNN; **G**: RetinaNet; **H**: YOLOv3; **I**: Ilastik; **J**: Fiji.

To better understand the sensitivity of the methods and the differences among the other used tools, we performed Spearman’s correlation analysis against the manual quantification (Fig 3). For images derived from rats, we observed a strong positive association when correlating the manual quantification with Faster R-CNN (R^2^ = 0.919; Fig 3A), RetinaNet (R^2^ = 0.935; Fig 3B), YOLOv3 (R^2^ = 0.942; Fig 3C) and Ilastik (R^2^ = 0.823; Fig 3D), and a moderate positive association with Fiji (R^2^ = 0.621; Fig 3E). In addition, as we validated our model on rats, mouse and NHP tissue, we performed an overall correlation analysis for the used tools. In this condition, we observed a strong positive association when comparing the manual quantification to Faster R-CNN (R^2^ = 0.847; Fig 3F), RetinaNet (R^2^ = 0.809; Fig 3G), YOLOv3 (R^2^ = 0.912; Fig 3H), a moderate positive correlation with Ilastik (R^2^ = 0.635; Fig 3I) and a weak positive correlation with Fiji (R^2^ = 0.269; Fig 3J) [65].

### Robustness

**Fig 4:**
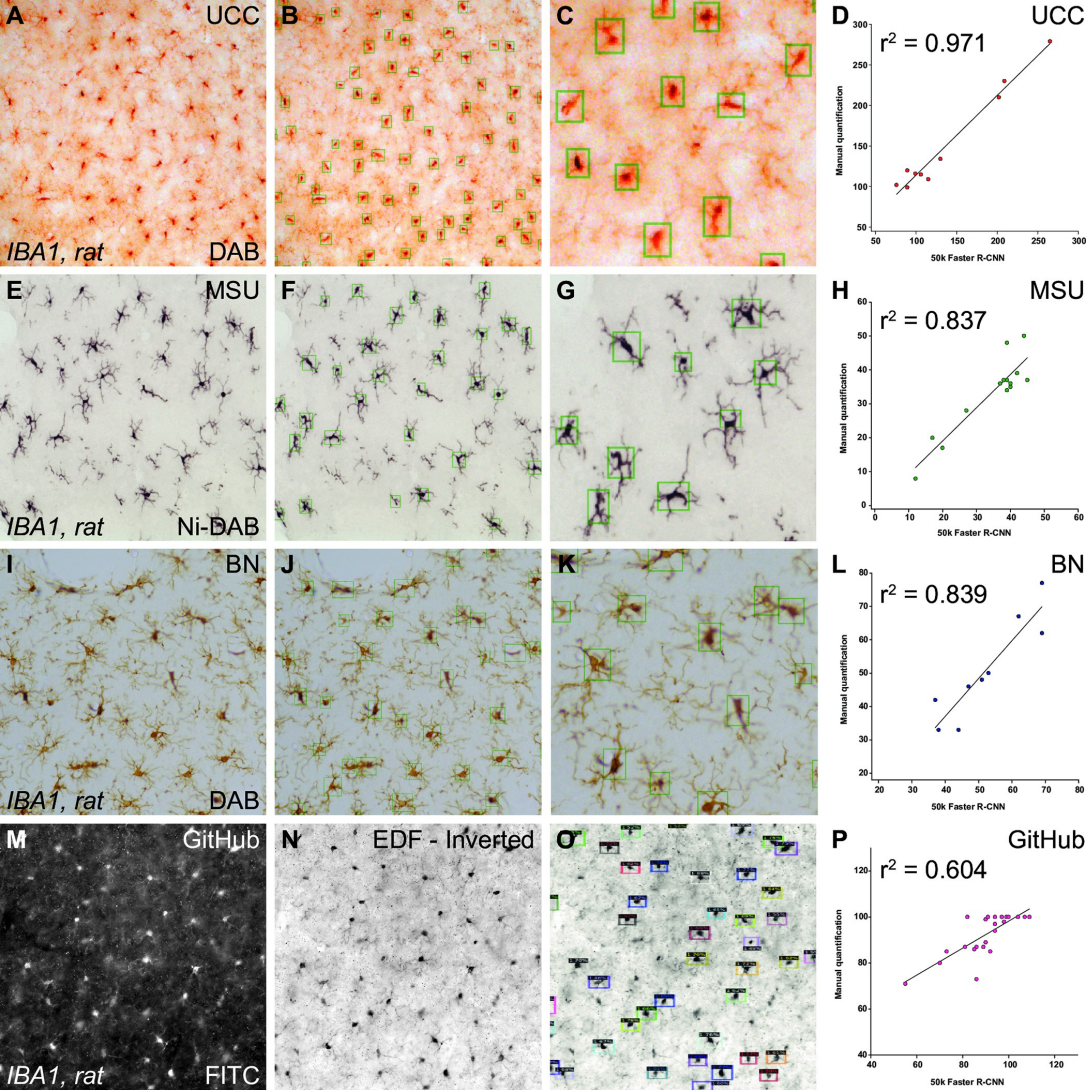
Robustness to images from other setups. Iba1-DAB immunolabelled brightfield images from three different laboratories (University College Cork, UCC (A-C); Michigan State University, MSU (E-G) and Barrow Neurological, BN (I-K). A representative image from the respective data set is presented in A, E, I and the processed image after object detection in B, F and J, respectively. A high magnification of detected cells is presented in C, G, and K. Correlations between the manually quantified images and the quantifications by the algorithm are presented in D, H and L. Iba1-FL-IHC images (M) downloaded from an online repository were inverted (N) and processed. A large proportion of visible cells can be detected by the algorithm (O). All manual quantifications correlated highly with the automated quantification (D, H, L, and P).

To check whether our algorithm is suitable for images/IHC processed tissue by other laboratories we asked three of our collaborators to test and validate our algorithm on their Iba1 immunolabelled tissue, respectively. The correlations of our CNN with the respective ground truth (manual quantifications) was r^2^ = 0.9714 (UCC; Fig 4A–4D), r^2^ = 0.8374 (MSU; Fig 4E–4H) and r^2^ = 0.8398 (BN; Fig 4I–4L), respectively. Although not an exhaustive list, this demonstrates that our method is suitable, with some tuning of the threshold and NMS parameters, for images generated from different IHC protocols and microscope settings. In a first attempt to extend the analysis to commonly used IF images we downloaded 24 images from a public Iba1-immunolabelled library (https://github.com/tkataras/ACCT-Data-Repository). These images were provided in grayscale with 2–3 individual focus layers. Using ImageJ we inverted the images, generated a stack from the individual layers and subsequently generated an EDF image (Sorbel projection) which we used for the analysis. As can be seen in Fig 4M–4P, the algorithm is able to detect the majority of cells in the image (r^2^ = 0.6042), although there is a substantial proportion of cells that are not detected (false negatives). In these and other instances where the performance of the algorithm is sub-optimal, further fine-tuning (i.e. training) will be necessary. For that purpose, we provide a step-by-step training guide (see materials and methods). Note that the images downloaded were different to our recommended settings. We used EDF images generated from 1μm z-stacks through the entire thickness of the section using an 20x objective whereas here we analysed images with only 2–3 focal planes taken at 10x.

**Fig 5:**
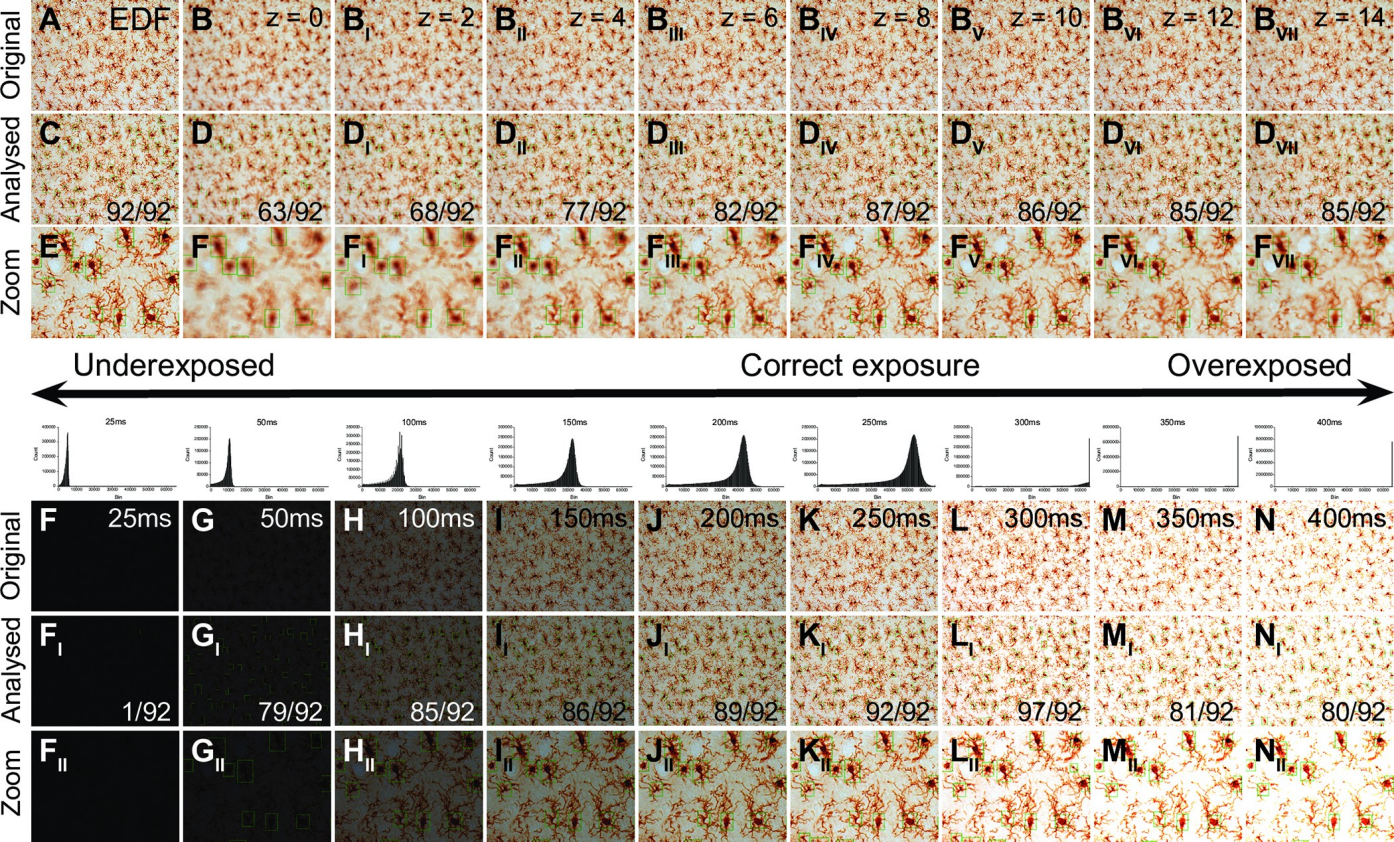
Optimal image settings for object detection of Iba1 immunolabelled cells in DAB. Original Iba1-DAB immunolabelled brightfield images from one animal (A-B_VII_) as EDF image of the entire z-stack (A) or as individual focal planes (B-B_VII_). The corresponding analysed image (C-D_VII_) and a high magnification of the analysed image (E-F_VII_) demonstrate that only the EDF image is able to identify the majority of immunolabelled cells in the stack correctly. The same z-stack is imaged under different exposure settings (25ms– 400ms) to utilise different parts of the dynamic range of the camera chip. The images are presented before processing (F-N) and after processing (F_I_-N_I_) as well as in high magnification (F_II_-N_II_). The histogram above the column displays the range of pixel values taken at the respective exposure setting.

Although we have demonstrated that our algorithm can provide a fast and reliable method to quantify microglial cells in thick tissue sections, the validity of the respective quantifications will depend on the quality of the histology. Here we image our samples using an 20x objective in colour in 1 μm z-steps through the entire thickness of the section and export the image as EDF file for further processing. Outside these parameters the performance of the algorithm will most likely be sub-optimal, for example when the image is taken in a single focal layer containing out of focus cells in the background (see Fig 5A–5E_viii_) or when the data is not spread out over the entire range of values possible during image acquisition at the bit-depth supported by the camera (i.e. using the camera’s dynamic range)(see Fig 5F–5O_viii_).

## Discussion

Current medical research needs unbiased standardised approaches to improve both experimental reproducibility between researchers, but also to facilitate translational research efforts from animal studies to clinical trials [66].

Unbiased quantification of cells number is a crucial tool in many pre-clinical and clinical studies. Classic approaches are based on unbiased stereological approximation using the optical fractionator principle [67]. Even if unbiased stereology is precise, it is very time-consuming and requires well trained user with specialized equipment as well as proprietary software. Furthermore, the stereology principle is based on a relative homogeneous distribution of cells within the area to be analysed, hence not all brain regions/nuclei lend itself to this form of analysis. Recent developments in computations, algorithm design, and GPU based computing have led to the exploration of machine learning in different domains of medicine and biological sciences, including in cell quantification [68–81]. In recent years various tools have been developed for characterization, detection, and classification of microglial and glial cells; all these tools unfortunately require one or more pre-processing steps (e.g., application of filters and threshold to enhance the structures of interest), as well as the use of several different software packages for image preparation and subsequent quantification [69, 82, 83].

The aim of our study was to develop a novel AI workflow for the detection of microglial cells, that limits the human manipulation and interference with the quantification process. Using our method, the user will need to acquire images of the target area with the microscope and directly process the files with the algorithm to obtain the microglial cell count. Depending on the processing of the tissue (IHC protocol) and microscopy (image quality/resolution), further training might be necessary. In the current work, we compared Faster R-CNN, RetinaNet and YOLOv3 object detection CNNs, trained on post-mortem tissues (DAB-IHC) following brightfield microscopy, to quantify microglial cells in relevant target structures. Faster R-CNN has high accuracy, but limited inference speed, while RetinaNet and YOLOv3 are single stage architectures, which directly give the bounding boxes coordinates and probability scores without a region proposal network. However, single stage architectures give less accuracy than the two stage architectures. To make the algorithm more competitive and to test its reliability, we also compared the networks with current available open-source software (Ilastik and Fiji), as well as with an unbiased researcher-blind manual quantification. In addition, to test the robustness of the model we examined the performance on 3 different mammalian species (mouse, rats and NHP) that are frequently used in preclinical research. We observed that neural networks–in all the conditions—were able to detect and perform an accurate quantification of microglial cells which was similar to the one obtained manually by an experienced user.

Although we did not observe any significant difference between the AI algorithms, we noticed a trend where YOLOv3 always slightly overquantified the number of microglial cells in all the settings; however, in the NHP specimens, YOLOv3 performed better than Faster R-CNN and RetinaNet, returning similar results to the manual quantification. With current knowledge and state of technology, we are unable to provide an explanation for this pattern [84]. In this work, we did not observe significant problems with false detection of cells neither during the testing phase of the algorithm or during the main validation study; this important issue has been avoided by always using two distinct and separate datasets for training protocols and validation protocols. Timewise, single image AI quantification was achieved within seconds, while the human user needed minutes, making this tool time and cost-efficient. On a biological level, the obtained results are in accordance with what is expected from these models and with previous published literature [39, 47, 85–87], confirming the reliability and sensitivity of the tool in the three species. Recent clinical and genetical studies have enfolded the role of microglial cells as an essential contributor in late onset Alzheimer’s disease [88, 89]. Hence, the need for reproducible, translational, and effective analysis of microglial images is warranted in the microglia research community.

Importantly, although the algorithms were trained on tissue obtained from rats and NHP, they were robust and returned precise values of microglial cells obtained from mouse tissue. In PD, the presence of h-aSYN is well known to induce an activation and increase of microglial cells, possibly due to its synergistic interplay with h-aSYN in the midbrain environment [90–92]. Various imaging [93, 94] and post-mortem [95–99] studies performed on pre-clinical models of PD as well as on humans samples showed the clear link between inflammation and the disease. In this work, AAV-9 mediated overexpression of h-aSYN produced an increase of IBA1^+^ cells in the ipsilateral midbrain as compared with the contralateral hemisphere; these results are in accordance with our previously published studies [39, 47, 100]. Interestingly, we observed a slight increase of IBA1^+^ cells in the ipsilateral midbrain of the AAV-noTG rats compared with the contralateral midbrain receiving no injection. This increase was however lower than that observed with h-aSYN overexpression. This slight increase in microglial cell number is most likely related to the surgical intervention.

The machine learning-based algorithms described here presents various advantages to manual quantifications or traditional stereological approaches. Firstly, the analysis of large dataset can be performed in short time. Secondly, computer-based quantifications minimize the human user bias during quantification and thirdly the method presented here is freely available and does not require subscription to proprietary software. Although the actual quantification is detached from direct human manipulation, there are several stages where human bias is introduced to machine learning approaches, such as the selection of training data (sampling bias, group attribution bias, prejudice bias, confirmation bias), the labelling patterns, data distribution and the model selection (algorithmic bias) itself. All of these parameters will introduce a bias into the models’ predictions, hence the bias from the researcher will affect the results of the prediction algorithm. As the current model is trained on a relatively small data set, there are most certainly biases hardwired into the algorithm.

The algorithms presented here, including the network weights and a step-by-step user guide allow users anywhere to take advantage of this type of high-throughput image processing. The main limitations of machine learning based approaches come from the quality of data/images used for the analysis. Tissue preparation and IHC is essential for a good and reliable analysis; it is crucial to have a selective labelling of microglial cells, with a well-performed DAB revelation that let the positive-labelled cells clearly stand from the background. On this regard, it is also important the thickness of the tissue, as ticker tissue might lead to partial antibody penetration and difficulties in finding the good contrast and crispness of single cells; in this study we used tissues with a thickness of 20 μm and 40 μm, and we did not observe any issue with antibody penetration. Another important factor is the microscopy image provided as a higher-resolution input image results in improved single cell detection and quantification. We provide the labelled training images, network weights and a step-by-step user guide online (https://gitlab.com/cell-quantifications/Microglia; https://www.kaggle.com/datasets/bnllund/microglia) which can be used for further fine-tuning of the model. The algorithm described here is functional and validated for overall quantification of microglial cells in the species and antibodies previously specified. Although several of our collaborators successfully implemented the CNNs on their images, the algorithms here will not be generalizable to all tissue types/settings. There is variation between laboratories in IHC approaches and the microscopy available which influences the final images that needs to be processed. Further fine-tuning until the level of accuracy required is achieved are easily implemented with the training guide that is uploaded in conjunction with the model on our GitLab page. In case of different applications (e.g., different species, antibody type/target, object classification) further AI training will be necessary. Future work will train an object-classifier to distinguish the activation status of microglial cells which will provide another level of depth for the understanding of the inflammation process. To conclude, we developed an algorithm for detection of microglial cells *in vivo*, and we tested the reliability of the method on 3 different mammalian species. Our results are comparable to the manual quantification (but obtained in a faster way), showing that the AI can overcome experimenter bias once the training period has been completed. The algorithm in the future might need refinement (e.g., additional training) to improve the detection or to apply the same tool to different specimens or type of cells.
